# Cancer-associated fibroblast-secreted exosomal miR-423-5p promotes chemotherapy resistance in prostate cancer by targeting GREM2 through the TGF-β signaling pathway

**DOI:** 10.1038/s12276-020-0431-z

**Published:** 2020-11-04

**Authors:** Guang Shan, Juan Gu, Daoping Zhou, Lingxun Li, Wei Cheng, Yueping Wang, Tian Tang, Xuedong Wang

**Affiliations:** 1 Department of Urology, RenMin Hospital of Wuhan University, Wuhan, 430060 Hubei China; 2 Department of Medical Laboratory Science, The Fifth People’s Hospital of Wuxi, Nanjing Medical University, Wuxi, 214000 Jiangsu China; 3 Department of Pathology, The Fifth People’s Hospital of Wuxi, The Medical School of Jiangnan University, Wuxi, 214000 Jiangsu China; 4 Center for Precision Medicine, Anhui No. 2 Provincial People’s Hospital, Hefei, 230041 Anhui China; 5 Department of Biology, College of Arts & Science, Massachusetts University, Boston, 02125 MA USA; 6 Department of Oncology, RenMin Hospital of Wuhan University, Wuhan, 430060 Hubei China

**Keywords:** Cancer, Cancer

## Abstract

Therapeutic failure in prostate cancer (PC) is believed to result from its unusually invasive and metastatic nature. Cancer-associated fibroblasts (CAFs) are essential in the tumor microenvironment. We intended to study the role of CAF-derived exosomes in the context of PC and the potential regulatory mechanism associated with miR-423-5p and GREM2. CAF-derived exosomes decreased the chemosensitivity of parental PC cells and enhanced the drug resistance of drug-resistant cells. PC-associated fibroblast-derived exosomes carrying miR-423-5p increased the resistance of PC to taxane by inhibiting GREM2 through the TGF-β pathway. Inhibition of the TGF-β pathway partially reversed the increased drug resistance in PC cells induced by CAF-derived exosomes. Inhibition of miR-423-5p enhanced the drug sensitivity of PC cells in vivo. We showed that CAF-secreted exosomal miR-423-5p promoted chemotherapy resistance in PC by targeting GREM2 through the TGF-β pathway. This study may allow the development of novel approaches for PC.

## Prostate cancer: Vesicles containing microRNA spur drug resistance

Connective tissue cells that surround prostate tumors secrete tiny RNA-filled sacs that make cancer less susceptible to chemotherapy. These findings, from a team led by Xuedong Wang from Nanjing Medical University in Wuxi, China, suggest that manipulating the contents of these nano-sized vesicles could help treat drug-resistant prostate cancer. The researchers isolated vesicles released by fibroblast cells that surround prostate tumors. Within these vesicles, known as exosomes, which serve as carriers of biomolecules that facilitate cell-to-cell communication, were molecules of a short regulatory microRNA that the authors showed could enhance the resistance of prostate cancer cells to drug therapies. Blocking the activity of this microRNA or its target pathways helped increase the sensitivity of prostate cancer cells to chemotherapy, both in cell culture and in mice.

## Introduction

Prostate cancer (PC) is the 2^nd^ most common cancer and the 5^th^ most common cause of death^1^. Statistics show that there are 1.6 million males with PC, and 366,000 males die from PC each year^2^. Many factors, including age, race, and family heritability, have been associated with PC development^3^. Based on the severity of this disease, current treatment approaches for PC include hormones, bone-directed treatment, therapeutic vaccines, radiation, and surgery^4^. Although these treatments can significantly delay or inhibit PC progression, chemotherapy resistance usually leads to death^5^. Cancer-associated fibroblasts (CAFs) are dominant in the tumor microenvironment because of their function in promoting tumor progression^6^. Additionally, CAFs act as regulators of the immune system by secreting transforming growth factor-β (TGF-β) and generating conditions under which antitumor immune responses are weakened^7^. Furthermore, CAF-derived exosomes seemed to be an important trigger of tumor progression^8^.

Exosomes are ideal targets for the treatment of cancer because of their small sizes and multifunctional effects on cells^9^. The exact effects of exosomes on the promotion or inhibition of PC have been discussed^10^. Exosomes carrying proteins, messenger RNAs (mRNAs), and microRNAs (miRs) can be transferred between cells^11^, and miRs can regulate gene expression through degradation or suppression of target miRNAs^12^. In addition, the effects of miRs on PC have been discussed, and exosomal miRs are regarded as markers for PC^13,14^. miR-423-5p could result in the occurrence of malignant types and temozolomide resistance in glioblastoma^15^ and promote autophagy of hepatocellular carcinoma cells^16^. One recent report showed that exosomal miR-423-5p could promote tumor growth and metastasis by targeting Sufu^17^.

A microarray-based analysis in the present study showed that GREM2 was implicated in PC. GREM2, a DAN family member, is directly associated with cancers via its interactions with bone morphogenetic proteins^18^. However, the role of GREM2 in PC and the effect of CAF-derived exosomes on chemotherapy resistance in PC remain unclear. Therefore, we intended to investigate exosomal delivery within the PC microenvironment with CAFs. The abovementioned literature prompted us to surmise that CAF exosomes combined with miR-423-5p may exert effects on PC via the GREM2 and TGF-β pathways.

## Materials and methods

### Bioinformatics analysis

The PC drug resistance chip GSE64012 was obtained through the GEO database (https://www.ncbi.nlm.nih.gov/) and contained 3 taxane-sensitive samples and 9 taxane-resistant samples. Using sensitive samples as a control, we used the R language “limma” package to analyze the differences to obtain significantly differentially expressed genes with |logFC | > 2 and *P* < 0.05 as the screening criteria. The differentially expressed genes were analyzed by KEGG pathway enrichment analysis (https://www.kegg.jp/kegg/) to obtain biological process entries for gene enrichment. The TargetScan database (http://www.targetscan.org/vert_72/) and the miRSearch database (https://www.exiqon.com/miRSearch) were used to predict the upstream miRNAs of the target genes, the EVmiRNA database (http://bioinfo.life.hust.edu.cn/EVmiRNA#!/browse) was used to obtain miRNAs that were highly expressed in extracellular vesicles (EVs), and then, the intersecting miRNAs were obtained. Finally, the StarBase database (http://starbase.sysu.edu.cn/index.php) was used to analyze the expression of target miRNAs in PC samples originating from TCGA and the correlation between miRNAs and target genes.

### Isolation of CAF-derived exosomes

Exosomes extracted from CAF culture medium were cultured in DMEM-F12 (Sigma-Aldrich, Merck KGaA, Darmstadt, Germany) with 10% Exo-fetal bovine serum (FBS) (FBS depleted of exosomes, SBI, Mountain View, California, USA) and 1× antibiotic-antimycotics (Life Technologies, Gaithersburg, MD, USA). After centrifugation, the supernatants were collected and added to sterile tubes containing ExoQuick-TC™ Exosome Isolation Reagent (SBI) with 2 mL of ExoQuick-TC™ solution for every 10 mL of medium, followed by agitation. Then, the tubes were stored at 4 °C O/N for at least 12 h. The next day, the exosomes were centrifuged at 1500*g* for 30 min and at 1500*g* for 5 min at 4 °C to obtain white/beige exosomal pellets.

CAFs were treated with miR-423-5p inhibitor or scramble inhibitor (10 nM, Tebu-Bio, San Diego, CA, USA) with Oligofectamine (Invitrogen, Carlsbad, CA, USA). Six hours later, the cells were washed, and the culture media were renewed with fresh DMEM-F12 1% A/A supplemented with 10% Exo-FBS. Following a 1-day incubation, culture media were harvested for exosome preparation. When incubating recipient cells (LN-CaP, 22Rv-1 or C4-2) with exosomes, we routinely included these cells in the medium at a concentration of approximately 1 × 10^9^ particles/mL.

### Immunofluorescence

CAFs were fixed, permeabilized, and labeled with rabbit anti-vimentin, α-SMA, and FAP mAb (Abcam), followed by amplification with FITC goat anti-rabbit antibodies and Alexa 488 goat anti-FITC antibodies (Abcam). Next, nuclei were stained with DAPI, and a laser scanning confocal microscope (FV1000, Leica) was used for observation.

### Characterization of exosomes

The morphology of the exosomes was observed using a transmission electron microscope (Japan, Hitachi 7650). In addition, particle size was determined using dynamic light scattering (Malvern Instruments, Malvern, UK).

### Cell treatment

The human PC cell lines LN-CaP, 22Rv-1 and C4-2 were provided by ATCC (Manassas, VA, USA). Docetaxel-resistant LN-CaP (LN-CaP/DTX) cells, taxane-resistant 22Rv-1 (22Rv-1/TAX) cells and bicalutamide-resistant C4-2 (C4-2/BCT) cells were prepared by continuous exposure of the parental drug-sensitive LN-CaP, 22Rv-1 and C4-2 cells to gradient concentrations of drugs (Sigma-Aldrich). All cells were cultured in RPMI 1640 medium with 10% FBS, 100 U/mL penicillin and 100 mg/mL streptomycin (Invitrogen) with 5% CO_2_ at 37 °C.

According to the instructions of the lentiviral packaging kit (Open Biosystems), lentivirus-carrying hsa-miR-423-5p or hsa-miR-negative control and hsa-GREM2 or empty vector was packaged in human embryonic kidney 293T cells and harvested from the supernatant. Stable cell lines were established by infecting 22Rv-1/TAX cells with lentivirus.

### Reveres transcription quantitative polymerase chain reaction (RT-qPCR)

Total RNA was isolated with TRIzol (Invitrogen) and reverse transcribed into cDNA using a Transcriptor First Strand cDNA Synthesis Kit (Toyobo, Osaka, Japan). Next, quantitative real-time PCR was performed on an ABI7500 system with a SYBR Green (Thermo Fisher Scientific, USA). The PCR system contained 10 µL of 2× real-time PCR buffer (including 3 mmol/L Mg^2+^, 0.2 mmol/L dNTP and SYBR Green I fluorescent dyes), forward and reverse specific primer sets (5 mmol/µL, 0.32 µL), Taq DNA polymerase (5 × 10^6^ U/L, 0.2 µL), cDNA template (2 µL), and RNase-free ultrapure water to reach a volume of 20 µL. GAPDH was used as the internal control of GREM2 and TGF-β. For quantitation of miR-155, U6 was used as the internal control. The relative expression was quantified by normalization using the 2^−^^ΔΔCt^ method. The primers are exhibited in Table 1.

**Table 1:** Primer sequences.

Sequences	Forward	Reverse
miR-423-5p	GCCTGAGGGGCAGAGAGC	CCACGTGTCGTGGAGTC
GREM2	TTTCCCTGTCCTTGTTCCTG	TGCACCAGTCACTCTTGAGG
TGF-β	CTCGCCGCGCTCTACCTACCTA	ATGAGCCATTCGCAGTTTCACTGTA
U6	GCGCGTCGTGAAGCGTTC	GTGCAGGGTCCGAGGT
GAPDH	AACGACCCCTTCATTGAC	TCCACGACATACTCAGCAC

### Western blot analysis

Western blot analysis was conducted as previously reported^19^. The following primary antibodies were used: anti-GREM2 (1:1000, ab228736, Abcam, NY, USA) and anti-TGF-β (1:1000, ab92486, Abcam), as well as horseradish peroxidase-conjugated secondary antibodies (1:1000, ab150117, Abcam), with anti-β-actin antibodies (ab179467, Abcam) as the control. The membrane was developed via the Super Signal West Pico Kit.

### *In vitro* cytotoxicity tests

LN-CaP, 22Rv-1 and C4-2 cells were cultivated at 8 × 10^3^ cells/well in 96-well plates for 4 h. Then, docetaxel (serial dilution, from 0 to 5 μM)/taxane (serial dilution, from 0 to 10 μM)/bicalutamide (serial dilution, from 0 to 10 μM) was added to the cells for incubation for 72 h. Docetaxel, taxane and bicalutamide were purchased from Sigma-Aldrich Company (USA).

### Cell Counting Kit-8 (CCK-8) assay

LN-CaP, 22Rv-1 and C4-2 cells (2.5 × 10^3^ cells) were added to 96-well plates and treated with 10 μL CCK-8 (Dojindo, Japan) for 2 h. The proliferation rates were evaluated at 0, 24, 48, and 72 h.

### Colony formation assay

A colony formation assay was utilized to measure the colony formation ability and the chemosensitivity of the PC cells to docetaxel, taxane and bicalutamide. Cells (1 × 10^3^ cells/well) were seeded in 6-well plates and then exposed to docetaxel, taxane and bicalutamide for 24 h. Then, the cells were cultured for 8 d at 37 °C. Next, the cells were fixed in 10% formaldehyde for 40 min and stained in 0.1% crystal viola for 20 min. Finally, the colonies were counted.

### Flow cytometry

Cell apoptosis was detected using a FITC Annexin V Apoptosis Detection Kit (BD Biosciences, San Jose, CA, USA)^20^. Apoptotic cells were detected with a fluorescence-activated cell-sorting flow cytometer (BD Biosciences).

### Hoechst 33258 staining

After treatment with chemotherapeutic agents for 48 h, the Hoechst 33258 staining kit (Life, Eugene, OR, USA) was applied to observe the apoptotic cells. Each assay was carried out at least three times.

### RNA pulldown

RNA pulldown assays were performed to determine the miR-423-5p and GREM2 interaction as previously described^21^.

### Luciferase reporter gene assay

The reverse complementary sequence of miR-423-5p was synthesized and cloned to construct psiCHECK2-423. Likewise, the 3′-UTR of GREM2 containing the miR-423-5p binding site was amplified and cloned to construct psiCHECK2-GREM2. Then, the psiCHECK2 vector containing a second reporter gene (firefly luciferase) was utilized for endpoint lytic assays. Lipofectamine 2000 (Life Technologies) was applied for reporter transfection. After 48 h, luciferase activity was measured.

### Xenograft tumors in nude mice

BALB/c-nu mice (5 weeks) were randomly assigned to 4 groups (*n* = 6). Parental 22Rv-1 and 22Rv-1/TAX cells (1 × 10^7^ cells/0.1 mL of PBS) were injected subcutaneously into the left and right flanks of nude mice. One week later, taxane was injected around the tumors on the 12^th^, 15^th^, and 18^th^ days. When the tumors were visible, the following treatments were performed: (1) inhibitor control and (2) miR-423-5p inhibitor in parental 22Rv-1 and 22Rv-1/TAX subcutaneous mice. The mice were sacrificed on the 35th day, and the tumor volume and weight were measured.

### Immunohistochemistry

Serial Section (4 μm) were made for immunohistochemistry for GREM2 (ab228736, Abcam) and Ki67 (ab15580, Abcam)^22^. Five regions were randomly selected to observe the area and the intensity of positive staining by ImagePro Plus 5.1 software, and the average value was obtained to assess the protein levels.

### Statistical analysis

All experiments were performed 3 times. Data are presented as the mean ± standard deviation (SD). Statistical analysis was performed with GraphPad Prism 8 software. The *p*-values were obtained using an unpaired *t* test, one-way or two-way analysis of variance (ANOVA), followed by Sidak’s multiple comparison test or Tukey’s multiple comparison test. *P* < 0.05 was regarded as statistically significant.

## Results

### Identification of CAFs and CAF-derived exosomes

**Fig. 1:**
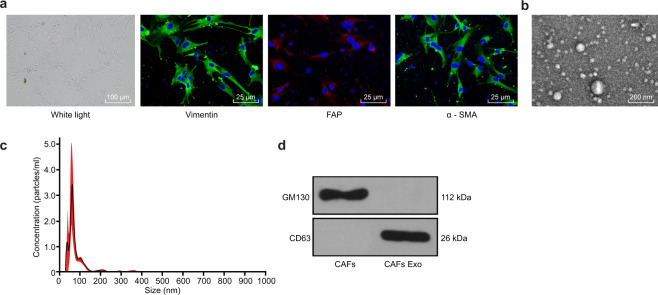
Characteristics of cancer-associated fibroblasts and isolation of exosomes. **a** Representative morphology of CAFs (scale bar, 200 μm) and immunofluorescence staining identification of CAFs using antibodies against Vimentin, α-SMA, and FAP (scale bar, 100 μm). **b** Transmission electron microscopy showing exosomes isolated from the CAFs (scale bar, 100 nm). **c** Nanoparticle tracking analysis of the CAF-derived exosomes (represented as size vs. concentration). **d** Western blot analysis of the exosome marker CD63 and the Golgi matrix protein GM130 in exosome-enriched conditioned medium.

We first observed that the cultured cells had a long spindle shape, multilayered growth and a disordered arrangement at a certain density, which is a typical cell morphology of CAFs. Then, we identified the CAF surface markers α-SMA and FAP and the fibroblast marker Vimentin by immunofluorescence. α-SMA, FAP and Vimentin were positive in the isolated and cultured cells, suggesting the successful isolation of CAFs (Fig. 1a). Subsequently, to investigate the functions of the CAF-derived exosomes, we isolated exosomes from the CAFs, and the CAF-derived exosomes were identified by transmission electron microscopy, nanoparticle analysis and Western blot analysis. The transmission electron microscopy results showed that the exosomes were round or oval membrane vesicles with basically the same shape (Fig. 1b). From the results of nanoparticle analysis, we observed that the diameter of the exosomes was approximately 90 nm, and most exosomes had a diameter of 30–150 nm (Fig. 1c). Western blot analysis revealed that CAF-derived exosomes were positive for CD63 but negative for GM130, indicating the successful isolation of CAF-derived exosomes (Fig. 1d).

### CAF-derived exosomes promoted drug resistance in PC cells

**Fig. 2:**
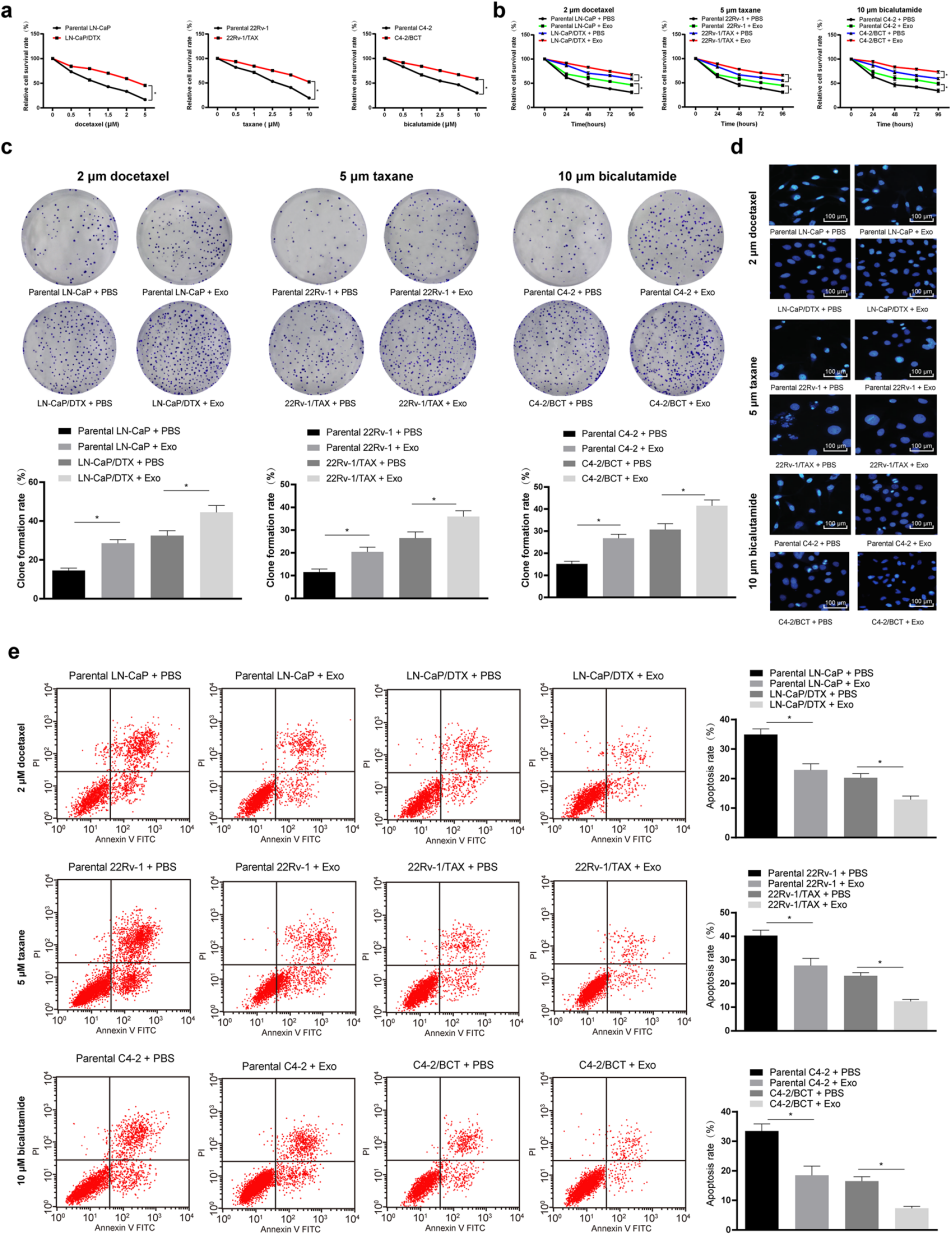
CAF-derived exosomes promoted drug resistance of prostate cancer cells. **a** Docetaxel-resistant LN-CaP (LN-CaP/DTX) cells, taxane-resistant 22Rv-1 (22Rv-1/TAX) cells and bicalutamide-resistant C4-2 (C4-2/BCT) cells were exposed to gradient concentrations of drugs (Sigma-Aldrich, St Louis, MO, USA) to generate drug-resistant prostate cancer cells, as shown by CCK-8 assays. Then, the parental or drug-resistant prostate cancer cells were treated with 20 μg/mL CAF-derived exosomes (Exo group) or PBS as a negative control (PBS group) for 24 h. Continuously, CAF-derived exosome-treated parental or drug-resistant prostate cancer cells were exposed to 2 μM docetaxel, 5 μM taxane and 10 μM bicalutamide for 2 h. Then, CCK-8 assays (**b**), colony formation assays (**c**), Hoechst 33258 staining (**d**) and flow cytometry (**e**) were performed to determine the effectiveness of the CAF-derived exosomes. Data are expressed as the mean ± standard deviation. One-way ANOVA and Sidak’s multiple comparisons test were used to determine statistical significance or two-way ANOVA and Tukey’s multiple comparisons test were used, *P < 0.05. Three independent experiments were performed.

To explore the effect of CAF-derived exosomes on drug resistance in PC cells, we first verified the successful construction of LN-CaP/DTX, 22Rv-1/TAX- and C4-2/BCT-resistant cell lines by CCK-8 assays (*P* < 0.05, Fig. 2a). Subsequently, the exosomes derived from the CAFs were added to the parent LN-CaP, 22Rv-1 and C4-2 cells and the corresponding drug-resistant cells at a dose of 20 μg/mL for culture for 24 h, and then, the cells were treated with 2 μM docetaxel, 5 μM taxane and 10 μM bicalutamide for 2 h. Next, CCK-8 assays, colony formation assays and flow cytometry were carried out and indicated that compared with the parental cells without treatment, the parental cells treated with the CAF-derived exosomes showed enhanced proliferation and colony formation and a decreased apoptosis rate. Compared with the drug-resistant cells without treatment, the drug-resistant cells treated with the CAF-derived exosomes showed enhanced proliferation and colony formation and a decreased apoptosis rate (*P* < 0.05, Fig. 2b–e). These results demonstrated that the CAF-derived exosomes decreased the chemosensitivity of the parental PC cells and enhanced the drug resistance of the drug-resistant cells.

### CAF-secreted exosomal miR-423-5p promoted drug resistance in PC

**Fig. 3:**
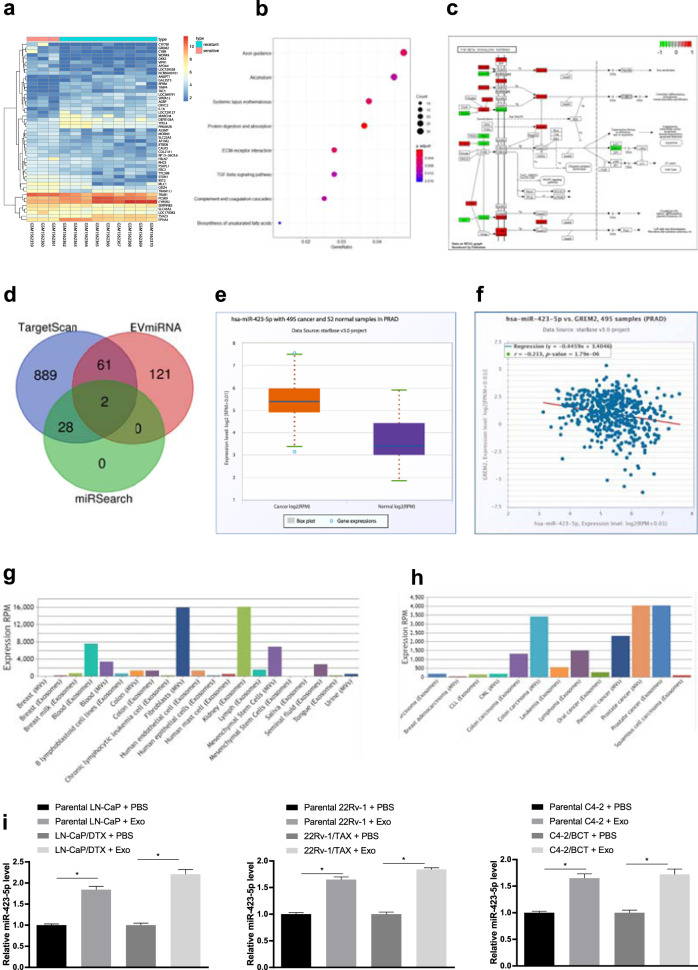
CAF-secreted exosomal miR-423-5p promoted drug resistance in prostate cancer. **a** The prostate cancer resistance chip GSE64012 was used; transverse coordinates represent the sample number, longitudinal coordinates represent the gene names, the left dendrogram shows gene expression clustering, and the upper right histogram shows the color gradation. **b** Enrichment analysis of KEGG pathways. The horizontal coordinates represent the GeneRatio, the vertical coordinates show the KEGG entry, and the right histogram is the color gradation. **c** Position and differential expression of differentially expressed genes in the TGF-β signaling pathway; red indicates genes that were highly expressed in the drug-resistant samples, and green indicates genes that were lowly expressed in the drug-resistant samples. **d** Prediction of miRNAs regulated upstream of GREM2. The three circles in the figure represent the prediction results of the two databases, and the miRNAs, the intermediate part of the EVmiRNA database, which were highly expressed in the fibroblasts microbubbles, represent the intersection of the three groups of data. **e** Expression of miR-423-5p in prostate cancer samples from TCGA. Horizontal coordinates represent the sample types, and vertical coordinates represent expression. **f** Correlation analysis of miR-423-5p and GREM2 expression in prostate cancer samples from TCGA. **g**, **h** Expression of miR-423-5p in exosomes from different samples and exosomes from different tumors. Horizontal coordinates represent sample types, and vertical coordinates represent miRNA expression. **i** RT-qPCR was used to detect the expression of miR-423-5p in the prostate cancer cells treated with CAF-derived exosome, with normalization to U6. Data are expressed as the mean ± standard deviation. One-way ANOVA and Sidak’s multiple comparisons test were used to determine statistical significance or two-way ANOVA and Tukey’s multiple comparisons test were used, *P < 0.05. Three independent experiments were performed.

The PC drug resistance chip GSE64012 was obtained from the GEO database. The chip includes 3 taxane-sensitive samples and 9 taxane-resistant samples. Using sensitive samples as a control, we used the R language “limma” package to analyze the differences, and 1964 significant differentially expressed genes in the drug resistance samples were obtained with |logFC | > 2 and *P* < 0.05 as the screening criteria. Figure 3a shows the expression heat map of some of the significantly differentially expressed genes. To further elucidate the drug-related signaling pathways, we analyzed these differentially expressed genes by KEGG pathway enrichment analysis, and the results showed that some differentially expressed genes were enriched in the TGF-β pathway (Fig. 3b). The TGF-β pathway is an important regulatory pathway in tumor development^23,24^. In the TGF-β pathway, 20 differentially expressed genes were enriched (Fig. 3c). We noted that the GREM2 gene was upstream of the TGF-β pathway, and its expression in the drug-resistant PC samples was significantly decreased, which indicated that it likely affected the resistance of PC to taxane chemotherapy by regulating TGF-β. Tumor-related fibroblasts influence the resistance of tumor cells to chemotherapy^25^. To further elucidate the upstream regulatory mechanism of GREM2, we predicted the upstream miRs of GREM2 using the TargetScan database and the miRSearch database. Furthermore, highly expressed miRs in fibroblast microvesicles were obtained from the EVmiRNA database. After determining the intersection of the predicted results and the highly expressed miRs in fibroblast microvesicles, we found that miR-423-5p and miR-382-3p were located in the intersection, and miR-423-5p showed a higher average expression value in fibroblast microvesicles than miR-382-3p (Fig. 3d). The average miR-423-5p expression was higher in the PC samples than in the normal samples, as shown by TCGA analysis (Fig. 3e). Correlation analysis was performed on the expression of miR-423-5p and GREM2 in TCGA, and we found that miR-423-5p was negatively correlated with the expression of GREM2 (Fig. 3f). In addition, miR-423-5p was highly expressed in fibroblast microvesicles and PC exosomes (Fig. 3g, h). Furthermore, miR-423-5p expression increased significantly in the PC cells after treatment with the CAF-derived exosomes (*P* < 0.05, Fig. 3i). These results suggested that the PC-associated fibroblast-derived exosomes carrying miR-423-5p increased the resistance of PC to taxane by inhibiting GREM2 through the TGF-β pathway.

### Inhibition of miR-423-5p attenuated the drug resistance of PC cells via CAF-derived exosome treatment

**Fig. 4:**
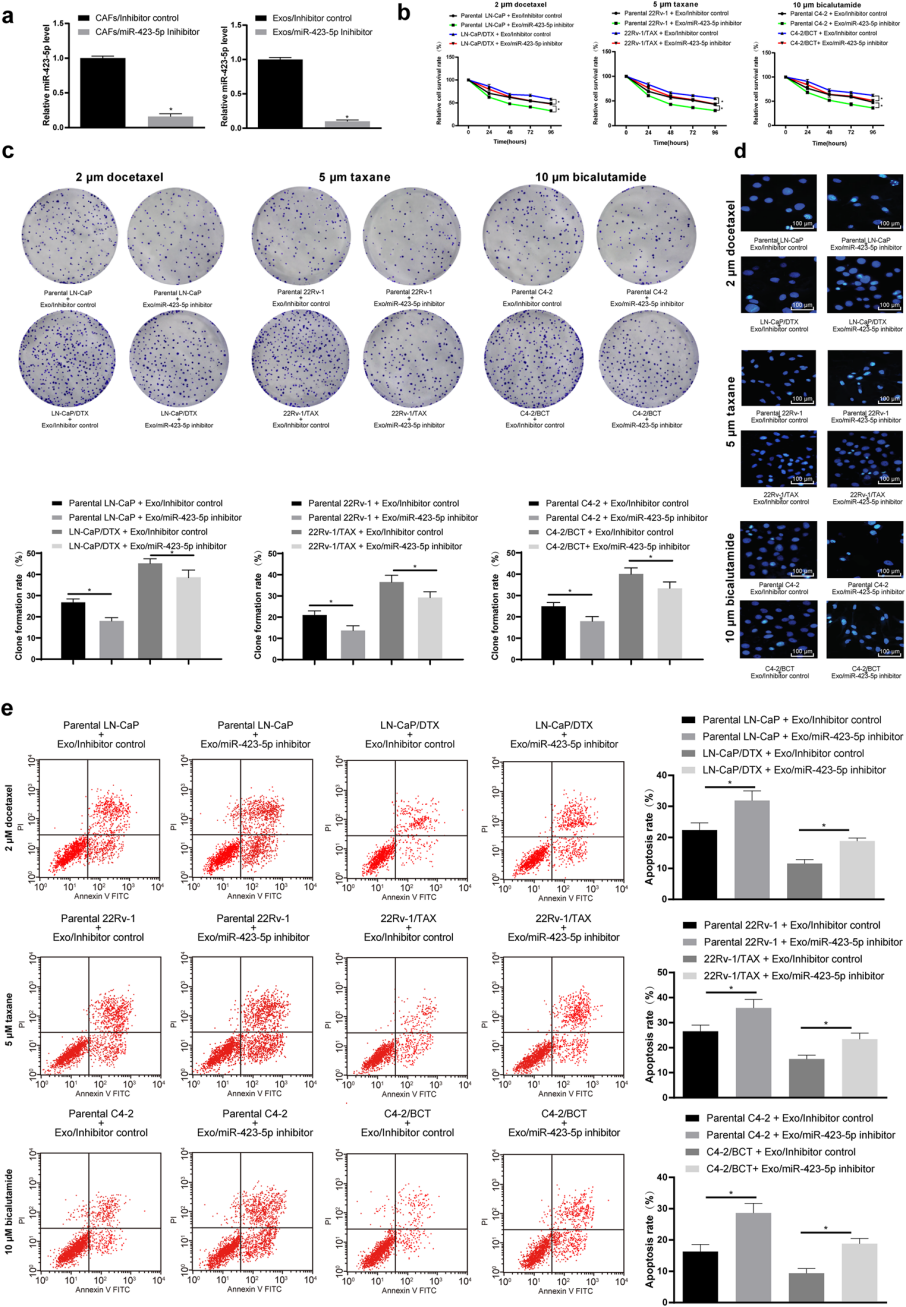
Inhibition of miR-423-5p attenuated the drug resistance of prostate cancer cells via CAF-derived exosome treatment. The miR-423-5p inhibitor was transfected into CAFs, while the inhibitor control served as the negative control. Then, exosomes (Exo/inhibitor control group and Exo/miR-423-5p inhibitor group) were extracted as described in the “Materials and methods” section. **a** RT-qPCR was performed to determine miR-423-5p expression in the CAFs and the exosomes. Continuously, CAF-derived exosome-treated parental or drug-resistant prostate cancer cells were exposed to 2 μM docetaxel, 5 μM taxane and 10 μM bicalutamide for 2 h. Then, CCK-8 assays (**b**), colony formation assays (**c**), Hoechst 33258 staining (**d**) and flow cytometry analysis (**e**) were performed to determine the effectiveness of the CAF-derived exosomes. Data are expressed as the mean ± standard deviation. One-way ANOVA and Sidak’s multiple comparisons test were used to determine statistical significance or two-way ANOVA and Tukey’s multiple comparisons test were used, *P < 0.05. Three independent experiments were performed.

To further evaluate the effect of CAF-secreted exosomal miR-423-5p on the drug resistance of PC cells, we inhibited miR-423-5p expression in CAFs through transfection of a miR-423-5p inhibitor (transfection of an inhibitor control was regarded as a control) and then extracted the exosomes. After miR-423-5p intervention, miR-423-5p expression in the CAFs and the CAF-Exos decreased significantly, as determined by RT-qPCR (*P* < 0.05, Fig. 4a). In addition, we treated the parental cells and the drug-resistant cells with the extracted exosomes (after transfection of the miR-423-5p inhibitor in the CAFs) (Exo/inhibitor control and Exo/miR-423-5p inhibitor). Then, CCK-8 assays, colony formation assays and flow cytometry were carried out and indicated that compared with the Exo/inhibitor control treatment, the Exo/miR-423-5p inhibitor treatment could significantly inhibit proliferation and colony formation of the parental cells and the drug-resistant cells while increasing the apoptosis rate, suggesting that the decrease in exosomal miR-423-5p partially reversed the decrease in drug sensitivity and the increase in resistance of PC cells induced by CAF-Exo treatment (*P* < 0.05, Fig. 4b–e).

### CAF-secreted exosomal miR-423-5p promoted chemotherapy resistance of PC by targeting GREM2

**Fig. 5:**
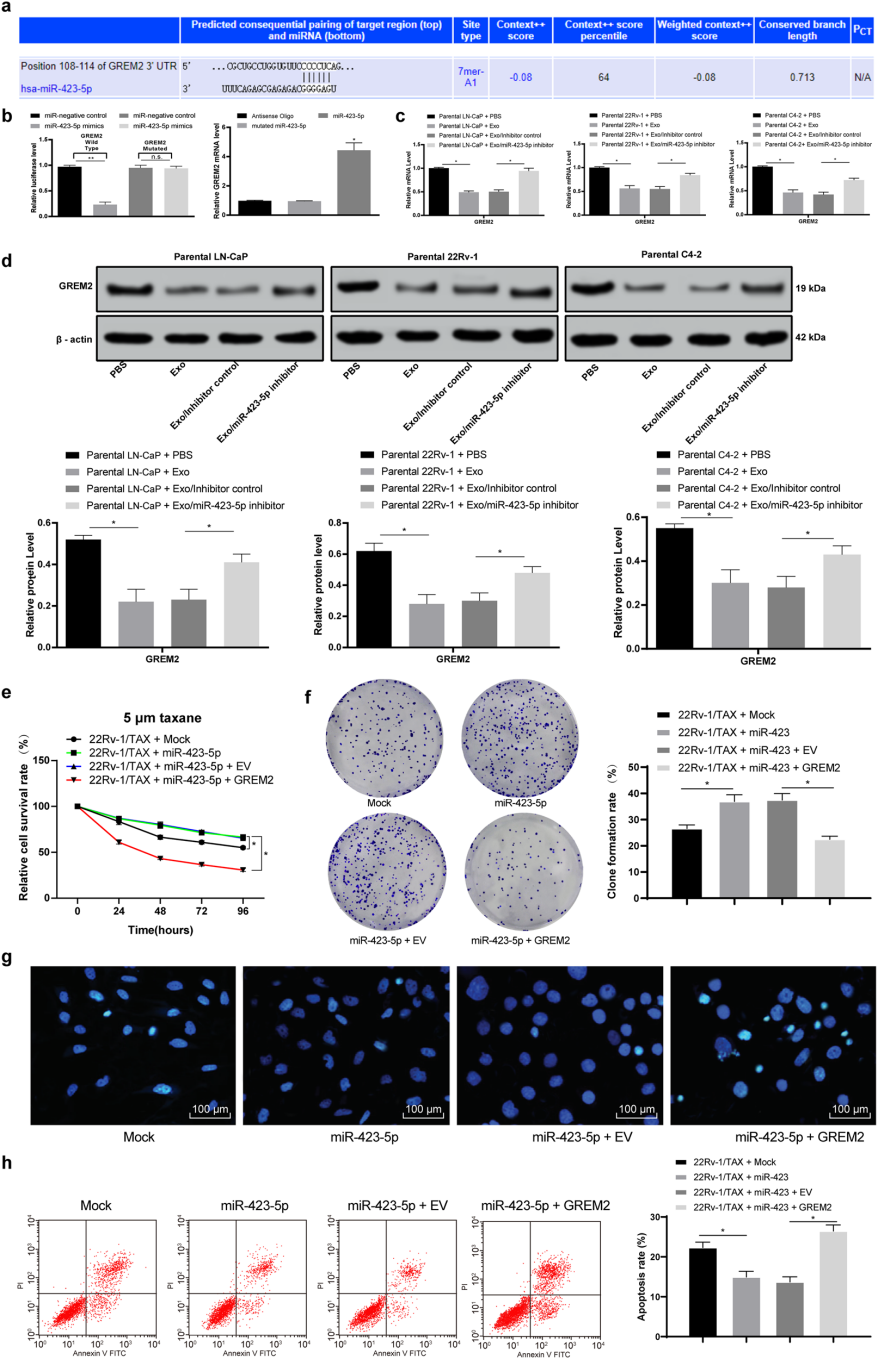
CAF-secreted exosomal miR-423-5p promoted chemotherapy resistance of prostate cancer by targeting GREM2. **a** TargetScan predicted that miR-423-5p could bind to GREM2 sequences. **b** Luciferase reporter plasmid containing GREM2-WT or GREM2-Mut was transfected into 293T cells together with miR-423-5p in parallel with a miR-NC plasmid vector. The enrichment of miR-423-5p on GREM2 was detected by RNA pulldown-qPCR assays relative to antisense oligos. Furthermore, RT-qPCR and Western blot analysis were performed to validate the GREM2 mRNA (**c**) and protein (**d**) levels. miR-423-5p was transfected into 22Rv-1/TAX cells, and a GREM2 expression vector was transfected. The miR-negative control (mock group) and empty vector (EV group) served as the negative control. Cells were exposed to 5 μM taxane for 2 h. Then, CCK-8 assays (**e**), colony formation assays (**f**), Hoechst 33258 staining (**g**) and flow cytometry analysis (**h**) were performed. The expression of miR-423-5p was normalized to that of U6, while the GREM2 mRNA level was normalized to that of GAPDH. Data are expressed as the mean ± standard deviation. One-way ANOVA and Sidak’s multiple comparison test were used to determine statistical significance or two-way ANOVA and Tukey’s multiple comparison test were used, *P < 0.05, **P < 0.01. Three independent experiments were performed.

To further explore the downstream mechanism of miR-423-5p, we designed a double-luciferase experiment based on the binding sequence of miR-423-5p and GREM2 in TargetScan, and the results confirmed that miR-423-5p could target GREM2. Then, RNA pulldown experiments verified that miR-423-5p could target the 3’-UTR sequence of GREM2 (*P* < 0.05, Fig. 5a, b). Subsequently, to verify the effect of miR-423-5p on GRAM2 expression, we determined the GREM2 levels in the PC cells treated with the CAF-derived exosomes, and the results showed that the GREM mRNA and protein levels in the PC cells treated with the CAF-derived exosomes were significantly decreased, while obvious changes were observed in the PC cells after exosomal miR-423-5p was decreased (*P* < 0.05, Fig. 5c, d). Additionally, to verify that miR-423-5p carried by the CAF-derived exosomes promoted chemotherapy resistance in prostate cancer by targeting GREM2, we selected 22Rv-1/TAX cells for functional rescue experiments. We overexpressed miR-423-5p in the 22Rv-1/TAX cells and found that the resistance to taxane was increased in the 22Rv-1 cells, as evidenced by the enhanced cell proliferation and colony formation and the decreased cell apoptosis rate. Furthermore, the resistance to taxane in the 22Rv-1/TAX cells decreased significantly after combined overexpression of miR-423-5p and GREM2 compared with overexpression of miR-423-5p only, as evidenced by the inhibited cell proliferation and colony formation and the increased cell apoptosis rate (*P* < 0.05, Fig. 5e–h).

### Inhibition of TGF-β pathway activity partially reversed the increase in drug resistance in PC cells induced by the CAF-derived exosomes

**Fig. 6:**
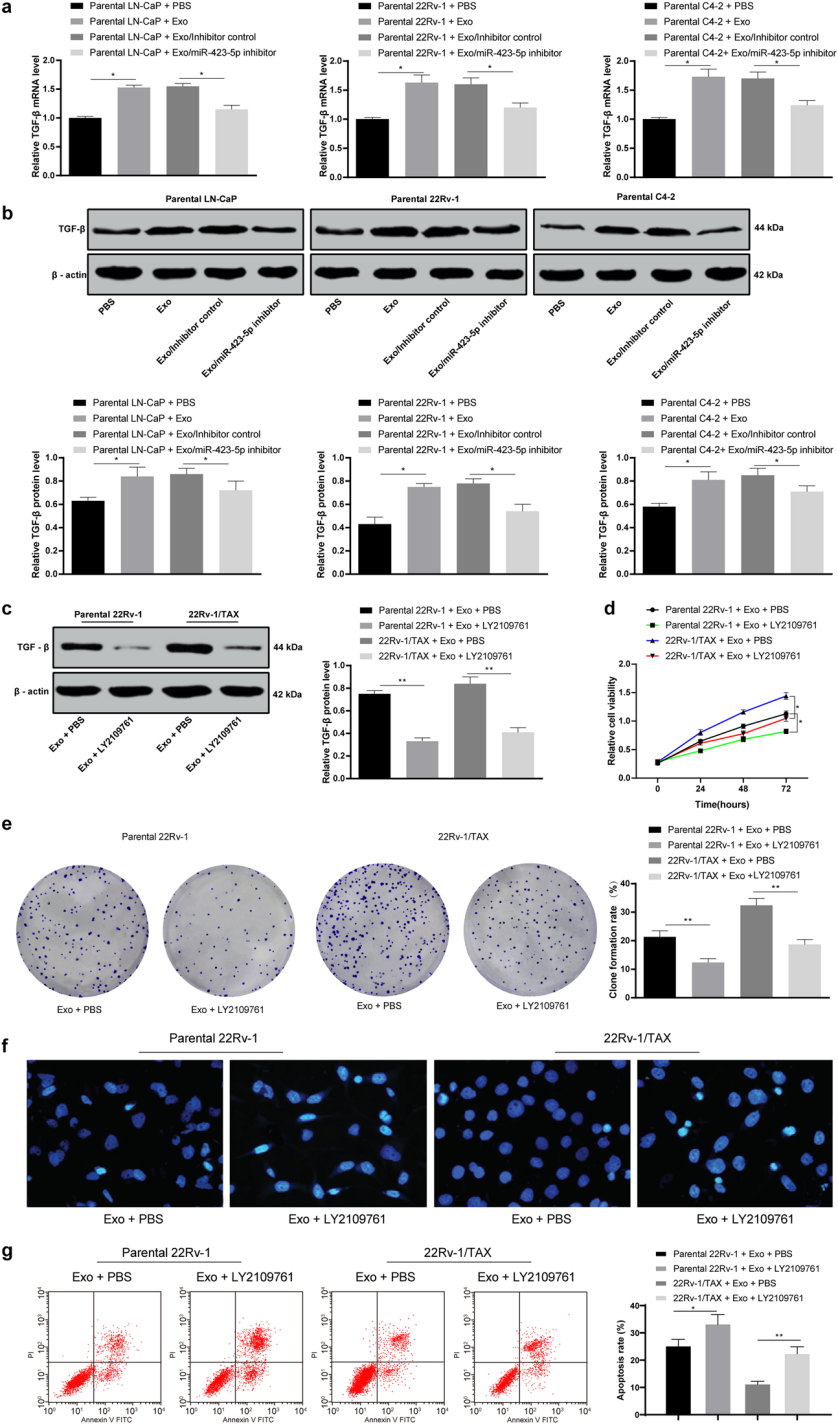
LY2109761 treatment partially reversed taxane resistance in prostate cancer cells via CAF-derived exosome treatment. RT-qPCR and Western blot analysis were performed to validate the TGF-β mRNA (**a**) and protein levels (**b**). LY2109761 is a TGF-β-specific inhibitor, and 15 μM LY2109761 was added to parental or taxane-resistant 22Rv-1 cells cocultured with the CAF-derived exosomes. Western blot analysis was performed to determine the TGF-β protein levels (**c**). Then, the cells were exposed to 5 μM taxane for 2 h. Then, CCK-8 assays (**d**), colony formation assays (**e**), Hoechst 33258 staining (**f**) and flow cytometry analysis (**g**) were performed to determine the effectiveness of LY2109761. Data are expressed as the mean ± standard deviation. One-way ANOVA and Sidak’s multiple comparison test were used to determine statistical significance or two-way ANOVA and Tukey’s multiple comparisons test were used, *P < 0.05, **P < 0.01. Three independent experiments were performed.

According to the results of Fig. 3, the TGF-β mRNA and protein expression in PC cells was detected. Exosome treatment increased the TGF-β mRNA and protein expression in the PC cells, while the TGF-β mRNA and protein expression in the PC cells increased after reducing exosomal miR-423-5p (*P* < 0.05, Fig. 6a, b). The above results further demonstrated that CAF-secreted exosomal miR-423-5p promoted the TGF-β pathway by targeting GREM2.

To further determine the role of the TGF-β pathway in the taxane resistance of PC cells after treatment with the CAF-derived exosomes, we selected 22Rv-1 and 22Rv-1/TAX cells for functional rescue experiments, and the TGF-β-specific inhibitor LY2109761 was used to treat the 22Rv-1 cells pretreated with the CAF-derived exosomes. Then, CCK-8 assays, colony formation assays and flow cytometry were carried out, and the results showed that TGF-β inhibition could partially reverse the increase in cell proliferation and colony formation as well as the decrease in the cell apoptosis rate, suggesting that TGF-β inhibition could suppress taxane resistance in the 22Rv-1 cells induced by the CAF-derived exosomes (*P* < 0.05, Fig. 6c−g).

### miR-423-5p inhibition increased the drug sensitivity of the PC cells in vivo

**Fig. 7:**
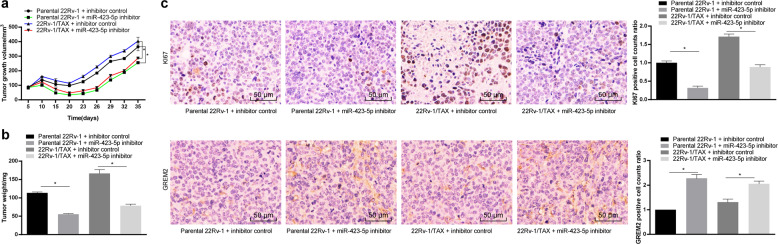
miR-423-5p inhibition suppressed taxane resistance of prostate cancer cells in vivo. Parental 22-Rv-1 and 22-Rv-1/TAX cells were inoculated subcutaneously into BALB/c nude mice at a dose of 5 × 10^6^ cells per mouse (*n* = 6 in each group). When the tumors were palpable, we treated the mice with the miR-423-5p inhibitor and the inhibitor control. Tumor growth was measured continuously every 5 days, and 20 days later, tumor growth was monitored every 3 days. Drug treatments were performed with taxane (10 mg/kg i.v.) on days 12, 15 and 18 after subcutaneous implantation. At 35 days postimplantation, the mice were euthanized via intraperitoneal injection of an overdose of pentobarbital sodium. **a** Tumor size. **b** Tumor weight. Tumor sections were obtained and stained with anti-GREM2 and anti-Ki67 antibodies. **c** Representative views of GREM2- and Ki67-positive tumor cells and quantification of immunostaining. Data are expressed as the mean ± standard deviation. One-way ANOVA and Sidak’s multiple comparison test were used to determine statistical significance or two-way ANOVA and Tukey’s multiple comparison test were used. *P < 0.05. Three independent experiments were performed.

The effect of miR-423-5p on the growth of the 22Rv-1 parental cells and the drug-resistant cells in vivo was evaluated by measuring the growth and weight of transplanted tumors in nude mice. The results showed that compared with the inhibitor control treatment, the miR-423-5p inhibitor treatment prevented tumor growth and reduced taxane resistance (*P* < 0.05, Fig. 7a) and decreased the tumor weight (*P* < 0.05, Fig. 7b). The positive expression of the proliferation marker Ki-67 detected by immunohistochemistry was significantly reduced (*P* < 0.05, Fig. 7c), indicating that inhibition of miR-423-5p expression could reduce the taxane resistance of PC cells in vivo. In addition, the results of GREM2 immunohistochemistry showed that the GREM2-positive cells increased after inhibiting miR-423-5p expression (*P* < 0.05, Fig. 7c). The targeted inhibitory effect of miR-423-5p on GREM2 was confirmed in vivo.

## Discussion

PC is the most common lethal malignancy^26^. miRNAs transferred by exosomes play important roles in the tumor microenvironment^27,28^. In the present study, we explored exosomal communication within the PC microenvironment. Our research demonstrated that miR-423-5p was upregulated in the CAF-derived exosomes and that miR-423-5p could promote chemotherapy resistance in PC by targeting GREM2 through the TGF-β pathway. These findings indicated that miR-423-5p-devoid exosomes derived from CAFs contribute to the malignant progression of PC.

Initially, our results revealed that GREM2 was substantially decreased in the drug-resistant PC samples, while miR-423-5p expression was increased in the PC samples. As shown in a previous study, the abnormal expression of miRs exerts different effects in multiple kinds of malignancies, including PC^14^. Previously, miR-423-5p expression was found to be upregulated in stable coronary artery disease, heart failure, acute myocardial infarction, and patients undergoing cardiac surgery^29,30^. Lin et al. proved that miR-423-5p was upregulated in human PC tissues and cells^31^. A previous study confirmed that GREM2 downregulation may be a marker in obese mice and humans^32^. However, no research has focused on its expression in PC. In addition, we found that miR-423-5p could target GREM2.

Our present study also showed that treatment with the CAF-derived exosomes decreased the chemosensitivity of the parental PC cells and enhanced the drug resistance of the drug-resistant cells. Accumulating evidence has shown that CAFs can stimulate invasion in breast cancer and non-small-cell lung cancer^33,34^. Additionally, CAFs were reported to advance tumor progression via specific communication within cancer cells. For example, Jing et al. demonstrated that epithelial ovarian cancer (EOC) cells could activate normal omentum via the TGF-β1 pathway and that CAFs could result in EOC cell invasion and adhesion^35^. In cancer cells, chemotherapeutic drugs can be delivered via exosomes^36^. Interestingly, studies have noted that oncogenic exosomes could accelerate invasion and drug resistance by delivering oncogenic DNA to normal cells^37–39^. Partly consistent with our study, previous studies also clarified that exosomes derived from CAFs could increase chemoresistance in colorectal and breast cancer cells^37,38^.

Furthermore, we found that the prostate cancer-associated fibroblast-derived exosomes carrying miR-423-5p increased the resistance of PC cells to taxane by inhibiting GREM2 through the TGF-β pathway. A recent study indicated that tumor-related fibroblasts can influence the resistance of tumors to chemotherapy^25^. CAFs known as myofibroblasts are induced and regulated by TGF-β^40–42^. Exosomes derived from PC cells can provide TGF-β to transform fibroblasts to myofibroblasts via activating the TGF-β/SMAD3 pathway^43,44^. It has also been proven that exosomes containing miRs serve as markers for PC^13^. Previous data suggested that CAF-derived exosomes could deliver miRs to neighboring epithelia, leading to increases in PC cells^45^. One recent report analyzed 29 paclitaxel-resistant PC cell-derived exosomal miRs and demonstrated that these exosomal miRs may result in PC chemoresistance^46^. Moreover, Lin H et al. confirmed that miR-423-5p inhibition suppressed PC cell growth and tumor volume^31^. CAFs can produce TGF-β, which in turn activates oncogenic signaling in tumor epithelial cells through the SMAD group of transcription factors^47^. Thus, we believe that blockade of the TGF-β pathway partially reversed the increase in drug resistance in PC cells induced by the CAF-derived exosomes.

In this study, we purified exosomes from CAFs and described their activities in vitro and in vivo. We further examined miR expression in exosomes. More importantly, we demonstrated that miR-423-5p was crucial in the chemoresistance of PC cells. This study indicated that CAF-derived exosomes could potentially be exploited as a therapeutic tool for PC.
